# Rapid synthesis of highly monodisperse AgSbS_2_ nanocrystals: unveiling multifaceted activities in cancer therapy, antibacterial strategies, and antioxidant defense

**DOI:** 10.3762/bjnano.16.145

**Published:** 2025-11-19

**Authors:** Funda Ulusu, Adem Sarilmaz, Yakup Ulusu, Faruk Ozel, Mahmut Kus

**Affiliations:** 1 Karamanoglu Mehmetbey University, Vocational School of Technical Sciences, Department of Crop and Animal Production, Karamanoğlu Mehmetbey University, 70200, Karaman, Turkey; 2 Department of Metallurgical and Materials Engineering, Faculty of Engineering, Karamanoğlu Mehmetbey University, 70200, Karaman, Turkey; 3 Karamanoglu Mehmetbey University, Faculty of Engineering, Department of Bioengineering, Karamanoğlu Mehmetbey University, 70200, Karaman, Turkey; 4 Recep Tayyip Erdogan University, Faculty of Engineering and Architecture, Department of Mechanical Engineering, 53020, Rize, Turkey; 5 Department of Chemical Engineering, Konya Technical University, Konya, Turkey

**Keywords:** AgSbS_2_ nanocrystals, antibacterial activity, antioxidant activity, cytotoxic activity

## Abstract

Nanocrystals (NCs) of silver antimony sulfide (AgSbS_2_) in the cubic phase were successfully synthesized using the hot-injection method. This study is the first to investigate the cytotoxic effects of these NCs on human breast adenocarcinoma (MCF-7), colon cancer cell lines (HT-29), and fibroblast cell lines (L929). Additionally, the antibacterial properties of the NCs against gram-positive (*Staphylococcus aureus* and *Bacillus subtilis*) and gram-negative (*Escherichia coli*) pathogenic bacteria were evaluated, along with their DPPH scavenging activities. The crystal structure of the synthesized NCs was elucidated through XRD analysis, revealing characteristic diffraction peaks corresponding to the (111), (200), (220), (311), and (222) planes of the AgSbS_2_ phase. TEM and SEM techniques were used to comprehensively characterize the NCs. The results showed that spherical NCs were predominantly formed, with an average diameter of approximately 32 ± 10 nm. Cytotoxicity studies demonstrated a significant inhibitory effect of the NCs, particularly on cancer cell lines (MCF-7 and HT-29), in a dose-dependent manner over a 24 h period. These findings highlight the potential of the NCs as anticancer agents. Furthermore, the synthesized NCs demonstrated potent antibacterial properties against the tested microorganisms and notable antioxidant effects by efficiently eliminating DPPH activity. This research highlights the potential of AgSbS_2_ NCs as versatile agents with applications in biomedical and environmental domains, including cancer therapy, antimicrobial strategies, and free radical neutralization.

## Introduction

Nanoscience serves as a unique platform to reveal new properties of substances through collaborative efforts with other fields (e.g., molecular chemistry, pharmaceutical science, applied health sciences, and engineering). In recent years, the integration of science and technology has been well orchestrated to address challenges in medicine and health sciences and to evaluate therapeutic aspects [1]. This integration has contributed to the development of a more efficient healthcare system, innovative nanomedical tools and advanced therapeutic approaches [2]. Currently, the assessment of nanotechnology’s impact on the health of both humans and animals, along with its potential in therapy, has become an imperative scientific consideration. Nanotechnology, which is multidisciplinary, is the synthesis of materials and particles with dimensions in the nanoscale range of approximately 1–100 nm; it has been considered important for the study of biological issues, and techniques and methods suitable for this approach have been developed and are still being developed by researchers [3–4]. Nanoparticles possessing distinctive physical, chemical, or biological attributes offer novel foundational elements for the design and development of devices and systems aimed at diagnosing and treating various diseases. Hence, nanoparticles or nanomaterials can be engineered to have various useful medical functions [4].

The assessment of the biological activity (e.g., antioxidant, antimicrobial, and anticancer) of nanomaterials has emerged as a crucial area of investigation in the field of nanoscience and nanotechnology. In many studies, chemically or biologically synthesized nanoparticles have a wide range of pharmacological activity ranging from antibacterial activity to tumor therapy activity [5–7].

The escalating global concern regarding antimicrobial resistance presents a considerable threat to public health. Researchers are persistently exploring alternative strategies to address this pressing issue and the critical demand for novel antimicrobial agents. Nanoparticle therapy is emerging as a prominent avenue toward that end [8]. Free radicals represent an important focus in pharmacological research. The severity of oxidative stress, defined as the imbalance between the formation of reactive oxygen/nitrogen species (ROS/RNS) and the activity of the organism’s antioxidative defense systems, is associated with various pathologies such as cancer, aging, and cardiovascular diseases [9].

Some anti-pathological responses of nanoparticles are mediated through increased production of ROS and induction of oxidative stress. This cascade of events leads to DNA breakage, heightened expression of death receptors, and ultimately culminates in apoptosis-driven cell death [10]. Therefore, nanoparticles can be considered as a medical agent in the treatment of various diseases that can be caused by free radicals, such as cancer, cardiovascular diseases, and diabetes [11–12].

Historically known especially for its antimicrobial properties, silver has been used since ancient times. Silver nanoparticles (AgNPs) are synthesized by different synthesis mechanisms; they are non-toxic to eukaryotic cells, including human cells, but highly toxic to prokaryotic cells including microorganisms such as bacteria, viruses, and fungi [13]. Therefore, silver-based nanoparticles have been the subject of many biomedical studies [14–18]. In a study conducted in this context; the effects of α-AgS nanoparticles produced using the fungus *Humicola* sp. in biomedical applications were investigated. Cancer experiments were carried out using breast cancer and Burkitt’s lymphoma cancer cells, while the biocompatibility tests of α-AgS nanoparticles were also conducted using human peripheral blood mononuclear cells (PBMCs) [18]. Additionally, these materials have also been used in various imaging applications for the detection of cancer cells. Ag_2_Te and Ag_2_S nanocrystals (NCs) were used in cancer imaging studies by Nieves and colleagues. In this study, computed tomography contrasts changes of NCs injected into mice were examined at 2 and 24 h [17]. In another study, the antibacterial effects of Ag, Ag_2_S, and Ag_2_Se NCs on gram-negative and gram-positive bacteria were investigated [15]. As a result of the literature review, biomedical applications of silver-based NCs were exemplified above. As can be seen from these studies, biomedical applications of AgSbS_2_ NCs have not been found. Therefore, biomedical applications of AgSbS_2_ NCs are presented to the literature for the first time with the present study.

## Materials and Methods

### Chemicals

Silver nitrate (AgNO_3_, ≥99.5%), *tert*-dodecylmercaptan (*t*-DDT, C_12_H_26_S), oleylamine (70%), antimony(III) chloride (SbCl_3_, ≥99.95%), 1-octadecene (ODE, 90%), and ethanol were obtained from Sigma-Aldrich. Toluene was obtained from VWR. 1-Dodecanethiol (1-DDT, C_12_H_26_S) was purchased from Alfa Aesar.

#### Rapid synthesis of AgSbS_2_ NCs

**Figure 1:**
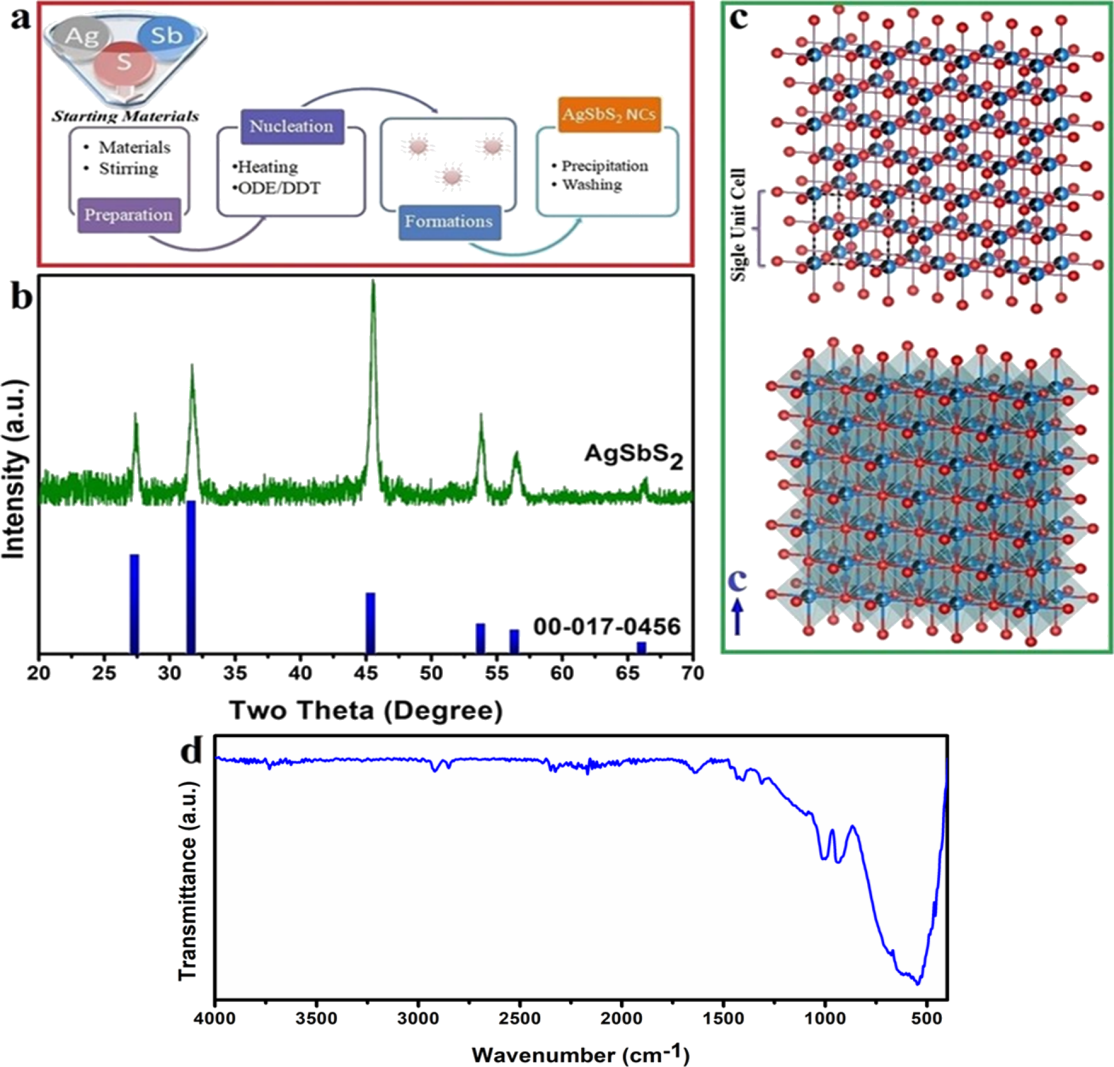
Synthesis procedure (a), XRD pattern (b), crystal geometry (c), and FTIR spectrum (d) of the synthesized NCs. Both crystal structure images were created by using the VESTA software [33].

Cubic-phase silver antimony sulfide NCs (AgSbS_2_ NCs) were synthesized by hot-injection method following a procedure adapted from previous studies [19–22]. The synthesis procedure is given below in Figure 1a. To synthesize AgSbS_2_ nanostructures, AgNO_3_ (0.5 mmol) and SbCl_3_ (0.5 mmol) were mixed with 20 mL ODE in a 25 mL two-necked, round-bottomed flask and they were evacuated at room temperature (25 °C) for 30 min under Ar flow. Then the reaction medium was started to be heated and when the reaction medium reached 160 °C, freshly prepared 1-DDT (0.52 mL) and *t*-DDT (3.52 mL) mixture was rapidly injected into the hot reaction medium under vigorous stirring. The synthesis was maintained at this temperature for 5 min, after which the reaction mixture was allowed to cool to room temperature. The obtained products were then precipitated using an ethanol/toluene mixture and subsequently collected by centrifugation (6500 rpm for 1 min).

### Antibacterial activities of AgSbS_2_ NCs

#### Disc diffusion assay

*Escherichia coli* ATCC^®^ 25922™ (gram-negative bacteria), *Staphylococcus aureus* ATCC^®^ 29213™, and *Bacillus subtilis* ATCC 6633 (gram-positive bacteria) were used for antibacterial assays. The antibacterial effectiveness of the synthesized NCs with different average sizes (15–20 nm) was evaluated by a disc diffusion assay [23]. In this study, overnight cultures of *S. aureus*, *B. subtilis*, and *E. coli* clinical pathogens were inoculated separately on Mueller–Hinton agar (MHA) plates. NCs were dissolved in 10% DMSO. Following, sterile discs (6 mm diameter) loaded with 10 µL (20 mg/mL) NCs were placed on the surface of MHA plates inoculated with 100 μL of a suspension comprising 10^8^–10^9^ CFU/mL of microorganisms and incubated for 24 h at 37 °C. In addition, ampicillin (10 mg/disc) and 10% DMSO were used as control for comparative effect. The zone of inhibition (ZOI) was measured and recorded. All experiments were performed in triplicate [24].

#### MIC and MBC measurements

The antibacterial activities of the synthesized NCs were evaluated using the broth microdilution method with resazurin as an indicator, following modified protocols from Elshikh et al. [25] and Veiga et al. [26]. Bacterial suspensions (*S. aureus, B. subtilis*, and *E. coli*) were prepared in Mueller–Hinton broth (MHB; Merck, Germany) at a density equivalent to 0.5 McFarland and adjusted to 10^5^–10^6^ CFU/mL. Sample stock solutions (10 mg/mL in 1% DMSO) were serially diluted in 96-well plates to final concentrations of 0.156–10 mg/mL. Each well received 10 µL of bacterial inoculum (final volume 100 µL), and plates were incubated at 37 °C for 24 h. Following incubation, bacterial viability was assessed using Alamar Blue^®^, with ampicillin as the positive control. The MIC was defined as the lowest concentration retaining a blue color, while the MBC was determined by subculturing non-turbid wells onto MHA plates and identifying the lowest concentration that yielded no visible bacteria colonies after 24 h.

### Cytotoxicity assays

#### Cell culture and Alamar Blue assay

HT-29 colon cancer, MCF-7 breast cancer, and L929 fibroblast cell lines were chosen as the experimental models in our in vitro study. Cell lines were cultured in DMEM (Dulbecco’s modified Eagle’s medium) supplemented with 10% FBS (fetal bovine serum), 1% penicillin/streptomycin solution (100 IU/mL/100 µg/mL) and 0.01% gentamicin. The cell lines to be tested were incubated in a humidified atmosphere containing of 95% air and 5% CO_2_ at 37 °C. Cell lines were grown to 80–90% confluency and then cells subcultured trypsinized with 0.25% Trypsin-EDTA [1].

Cytotoxic activities of NCs were performed by Alamar Blue^®^ assay (Sigma-Aldrich) on cell lines tested [27]. NCs were dissolved in DMSO (10 mg/mL) stock and the DMSO concentration in the cell culture medium was not more than 0.1%. Cells were seeded in 96-well plates at a density of 2 × 10^4^ cells/well, and the plates were incubated in a humidified incubator (95% air and 5% CO_2_) at 37 °C for 24 h. Triplicate of experiments were performed. After fixation, each well was washed twice with 1× PBS. Then, the cells were exposed to varying concentrations of NCs (12.5, 25, 50, 100, 200, and 400 µg/mL) and incubated at 37 °C for 24 h. Following the incubation period, Alamar Blue (10% v/v) was added to each well and incubated at 37 °C for 4 h. Absorbance was measured at 570 and 630 nm wavelengths using a spectrophotometric microplate reader (Multiscan Go, Thermo Fisher Scientific, USA). All data were expressed as a percentage of the control group (untreated cells; Equation 1). IC_50_ values were determined using GraphPad prism version 8 (GraphPad Software, San Diego, CA, USA).


[1] $$\text{percentage of cell viability}\left(\%\right)\;=\;\frac{{\text{OD}}_{570-630}\text{treatment}}{{\text{OD}}_{570-630}\text{control}}\;\times \;100\%$$

### Antioxidant activity of AgSbS_2_ NCs

#### DPPH radical scavenging activity assay

100 μL of 0.2 mM methanolic DPPH solution was combined with 50 μL of nanocrystal solution dissolved in methanol at varying concentrations (12.5–400 µg/mL). DPPH and nanoparticle mixed solutions were used as sample, while only DPPH solution was used as negative control and ascorbic acid was used as a positive control. The samples were kept in the dark at room temperature for 30 min of incubation, and the change in color was monitored, using a microplate reader configured to a wavelength at 517 nm [28–30].

The radical scavenging activity was calculated using Equation 2:


[2] $$\text{inhibition \%}=\frac{{\text{Ab}}_{\text{control}}-{\text{Ab}}_{\text{sample}}}{{\text{Ab}}_{\text{control}}}\times 100\%$$

In this formula, Ab_control_ refers to the absorbance of the DPPH solution, while Ab_sample_ represents the absorbance of the DPPH solution when combined with the sample.

#### FRAP assay

The ferric reducing antioxidant power (FRAP) of the synthesized NCs and their functionalized microcapsules was determined following the method described in [31]. The FRAP reagent was prepared by mixing 10 mM 2,4,6-tri(2-pyridyl)-*s*-triazine in 40 mM HCl, 20 mM FeCl_3_·6H_2_O, and 300 mM acetate buffer (pH 3.6) in a 1:1:10 (v/v/v) ratio. Methanolic sample solutions (12.5–400 μg/mL) were combined with the reagent (2.3 mL) in a total reaction volume of 3.0 mL and incubated at 37 °C for 30 min in the dark. Absorbance was recorded at 593 nm, with ascorbic acid as the standard and acetate buffer as the blank. Antioxidant capacity was expressed as micrograms of ascorbic acid equivalents per milliliter. All measurements were carried out in triplicate.

#### Statistical analysis

All experiments were performed in triplicate, and data were reported as mean ± standard deviation. Statistical analyses were conducted using one- or two-way ANOVA followed by Duncan’s post hoc test (SPSS v24.0, IBM Corp., Armonk, NY, USA). IC_50_ values were determined via nonlinear regression analysis using GraphPad Prism v8.0 (GraphPad Software, San Diego, CA, USA). The statistical significance was accepted at *p* < 0.05.

## Results and Discussion

### Structural and morphological characterization of AgSbS_2_ NCs

The crystal structure and phase of AgSbS_2_ NCs were investigated by XRD analysis. The obtained diffraction pattern and a schematic representation of the crystal structure are given in Figure 1. As can be seen from the XRD pattern, five dominant peaks at around 27.3°, 31.7°, 45.4°, 53.8°, and 56.4° were matched to the (111), (200), (220), (311), and (222) planes of AgSbS_2_ (JCPDS card No. 65-9810). Moreover, the obtained results were found to be consistent with previously reported literature. Although zeta potential was not experimentally measured in this study, it is generally accepted that the surface charge of NCs plays a critical role in their colloidal stability, dispersion behavior, and biological interactions [22,32].

In cubic cuboargyrite phase, there are 14 polyhedra with antimony and silver at the center (Figure 1c). Herein, a separate interaction exists between 6 sulfur atoms and silver and antimony atoms. Also, the obtained diffraction peaks are neat and intense, indicating the absence of other secondary phase peaks, and proving the successful synthesis of the desired structure.

FTIR analysis was carried out in the range of 4000–400 cm^−1^ to investigate the functional groups and chemical bond structures of the synthesized AgSbS_2_ NCs. The vibrational peaks observed in Figure 1d, appearing at 520–650 cm^−1^, were assigned to Ag–S bonds, whereas those in the 890–1040 cm^−1^ range were attributed to C=C–H bonds [34–35]. In addition, a weak peak centered around 1315 cm^−1^ corresponds to the C–H bending mode [36]. The characteristic peak around 1640 cm^−1^ was assigned to the O–H bending vibration, while the bands appearing in the 2800–3000 cm^−1^ range were attributed to the symmetric and asymmetric stretching vibrations of CH–H bonds [34–35].

**Figure 2:**
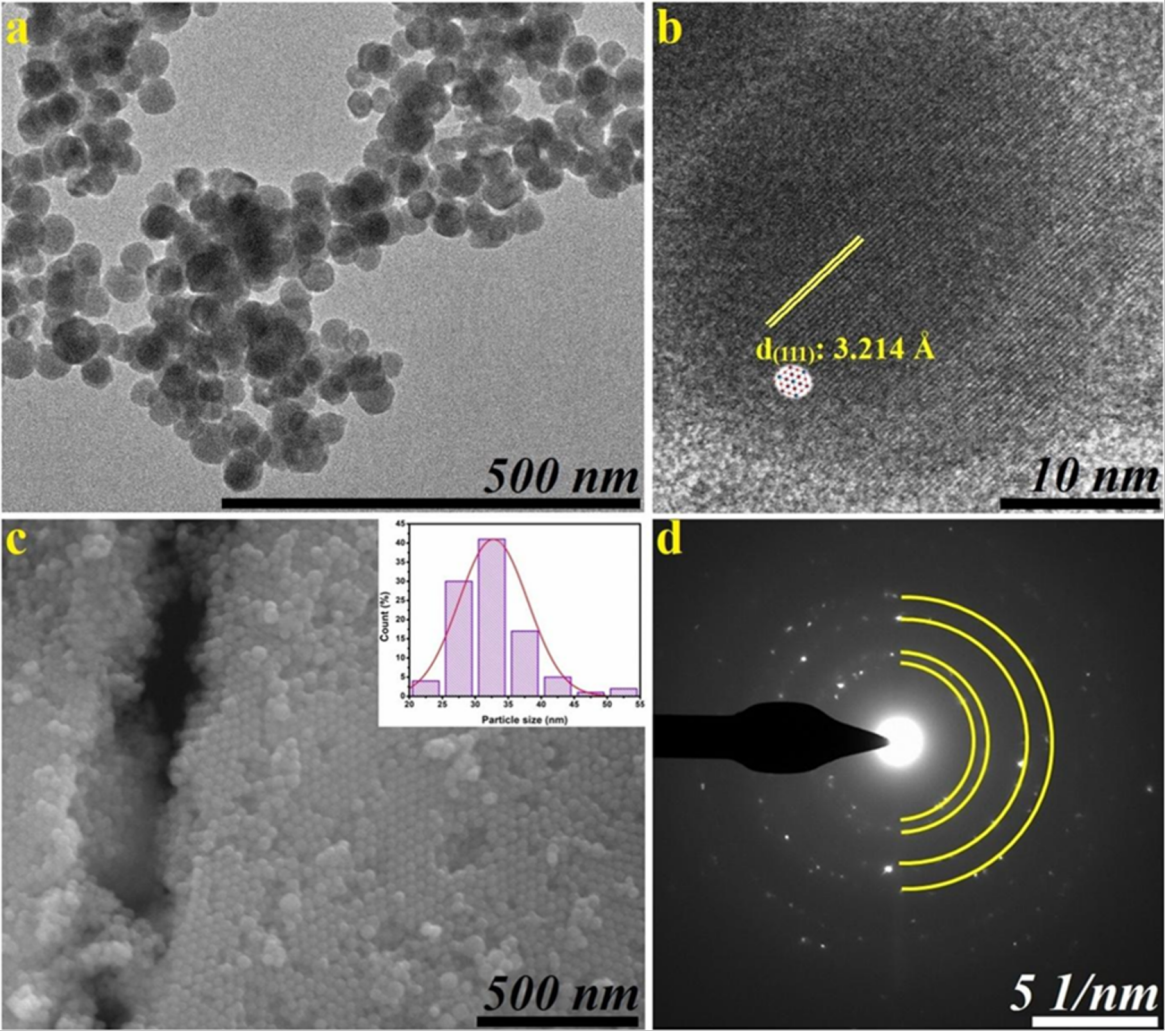
The TEM (a), HRTEM (b), SEM (c) images and SAED (d) patterns of AgSbS_2_ NCs.

The characterization of AgSbS_2_ NCs involved a comprehensive analysis using TEM and SEM techniques, as depicted in Figure 2. Within the SEM and TEM images presented in Figure 2a,b, predominantly spherical NCs are observed, measuring approximately 32 ± 10 nm in diameter. Notably, these findings were consistent across various imaging modalities. Analyzing the HRTEM image in Figure 2c revealed a lattice d-spacing of 3.214 Å, corresponding precisely to the (111) crystallographic planes. The SAED patterns in Figure 2d exhibited distinct, discontinuous diffraction rings with sharp points, indicating robust crystallization. Moreover, the presence of prominent peaks aligning with (220), (311), (420), and (511) planes confirmed alignment with XRD results. Furthermore, utilizing particle size distribution analysis, the crystallite size of average particles was estimated, as illustrated in the inset in Figure 2c. The particle size distribution graphs in this image unequivocally affirmed the successful attainment of the desired homogeneity.

### Biological applications

#### Antibacterial activity of AgSbS_2_ NCs

**Figure 3:**
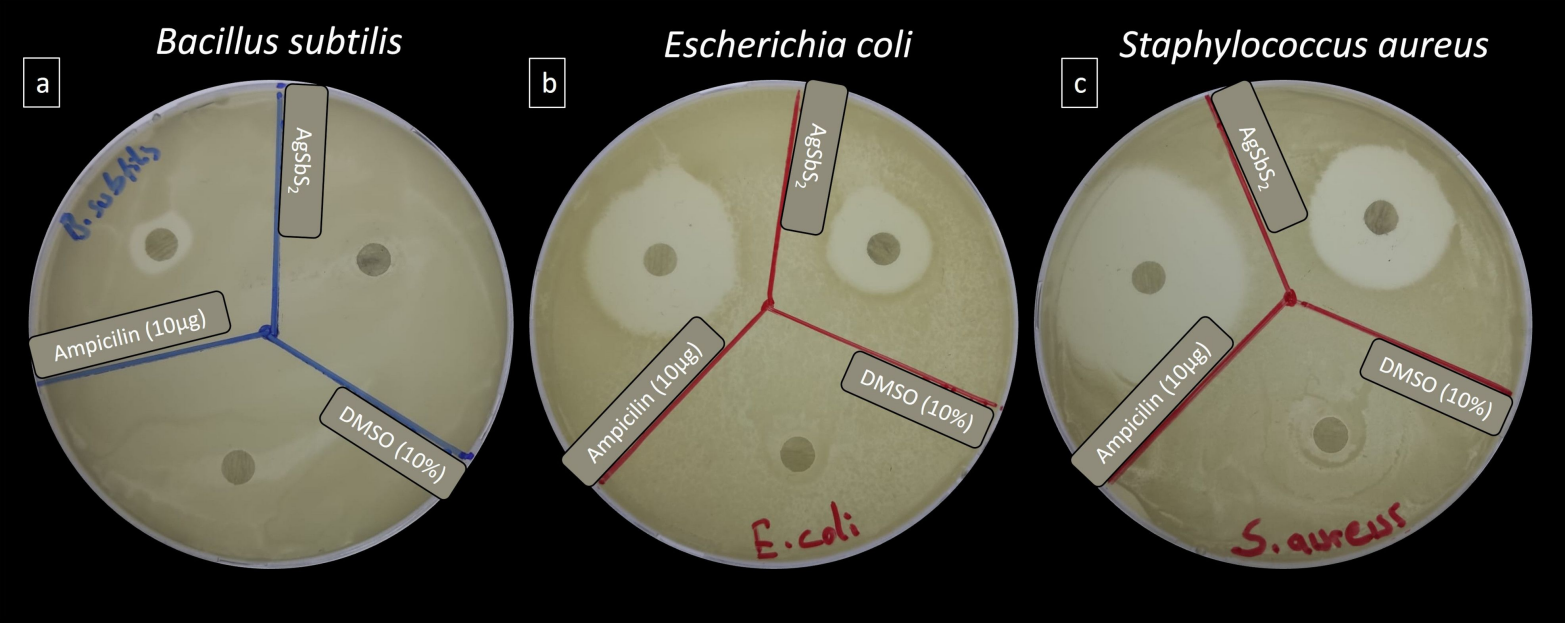
Zones of inhibition produced by AgSbS_2_ NCs against tested bacterial strains.

**Disc diffusion.** The antimicrobial activity of AgSbS_2_ NCs at a concentration of 10 mg/mL against gram-positive (*B. subtilis* and *S. aureus*) and gram-negative bacteria (*E. coli*) was investigated. The measured ZOI values are given in Table 1 and Figure 3. Following a 24 h incubation period, the antimicrobial efficacy against *B. subtilis*, *S. aureus*, and *E. coli* bacteria was scrutinized. The NCs exhibited substantial activity against both gram-positive and gram-negative bacterial strains, manifesting as distinct zones of inhibition ZOIs surrounding the discs. Compared to the established efficacy of ampicillin (10 µg/disc), the synthesized NCs displayed less pronounced antibacterial activity, as evidenced by a smaller ZOI. A maximum of ZOI diameter of 25.2 mm was recorded against *S. aureus* followed by 18.8 mm against *E. coli* (Figure 3). However, the antibacterial activity of the synthesized NCs proved to be relatively weak against *B. subtilis*, failing to produce any ZOI.

**Table 1:** MIC and MBC values of AgSbS_2_ NCs against the tested bacterial strains.^a^

	Median MIC/median MBC; MBC/MIC ratio		ZOI (mm)		
	positive control (μg/mL)	sample (10 mg/mL)	positive control (10 μg/disc)	negative control (10%)	sample (10 mg/mL)

bacterial strain	ampicillin	AgSbS_2_ NCs	ampicillin	DMSO	AgSbS_2_ NCs

*B. subtilis*	32.0/128.0; 4.0	5.0/ND; ND	7.2 ± 0.4	NZ	NZ
*S. aureus*	0.5/1.0; 2.0	0.5/1.0; 2.0	37.5 ± 0.5	NZ	25.2 ± 0.6
*E. coli*	6.0/12.0; 2.0	1.0/2.0; 2.0	29.5 ± 0.5	NZ	18.8 ± 0.2

^a^The data are presented as mean ± standard error (*n* = 3). ND: Not determined. NZ: No zone.

The direct interaction of nanoparticles with bacterial cells and the production of ROS, which causes DNA damage and denaturation of proteins close to the bacterial membrane, causes cell membrane damage [37–38]. In addition, the electrostatic force generated between the bacterial cells and the synthesized NCs causes distraction of the bacterial cell wall and cell disruption when the NCs enter into the cell [39]. Thus, induction of apoptosis in bacterial cells occurs, resulting in an inevitable antibacterial mechanism of action. The data shows that the synthesized NCs in the study can be used as an antimicrobial agent in various industrial fields and devices.

**MIC and MBC.** The MIC and MBC values for the bacterial strains determined in AgSbS_2_ NCs solution are shown in Table 1. The antibacterial susceptibility assays demonstrated distinct differences in the MIC and MBC values of AgSbS_2_ NCs across these strains.

Similarly to the disc diffusion assay findings, the strong inhibitory and bactericidal effect against *S. aureus* (MIC: 0.5 mg/mL; MBC: 1.0 mg/mL) of AgSbS_2_ NCs aligns well with previous reports that show that gram-positive organisms are often more susceptible to metal-based nanoparticles or nanocomposites [40], likely due to their simpler outer membranes compared to those of gram-negative species. The moderate effectiveness of AgSbS_2_ NCs against *E. coli* (MIC and MBC; 1.0 mg/mL and 2.0 mg/mL, respectively) also aligns with findings in nanoparticle research where the outer lipopolysaccharide layers of gram-negative bacteria act as a barrier, reducing the uptake of antimicrobial agents, thus, increasing the required inhibitory concentrations. Similar results have been observed for silver, ZnO, and other metal chalcogenide nanoparticles, which show higher MIC/MBC values for *E. coli* compared to *S. aureus* [41]. In size-dependent studies, larger nanoparticles tend to have higher MICs for *E. coli*, indicating that membrane permeability and nanoparticle size are key factors affecting antimicrobial strength [42].

Of particular interest is the high resistance exhibited by *B. subtilis*, which demonstrated an MIC of 5.0 mg/mL and an MBC that exceeded the highest tested concentration. This finding indicates that, for *B. subtilis*, AgSbS_2_ NCs function primarily as bacteriostatic agents at the concentrations examined, rather than as bactericidal agents. Such behavior is consistent with literature reports in which *Bacillus* species (due to their strong cell walls and sporulation capabilities) require significantly higher concentrations of nanoparticles for killing activity [43].

Taken together, the two antibacterial tests indicate that AgSbS_2_ NCs, with their low MIC/MBC values against *S. aureus*, have strong potential in treating infections dominated by this pathogen. However, their relatively weaker activity against *E. coli* and *B. subtilis* suggests that optimization may be necessary to broaden their spectrum through surface modification, particle size reduction, or combination with other antibacterial agents.

#### Cytotoxicity of AgSbS_2_ NCs

**Figure 4:**
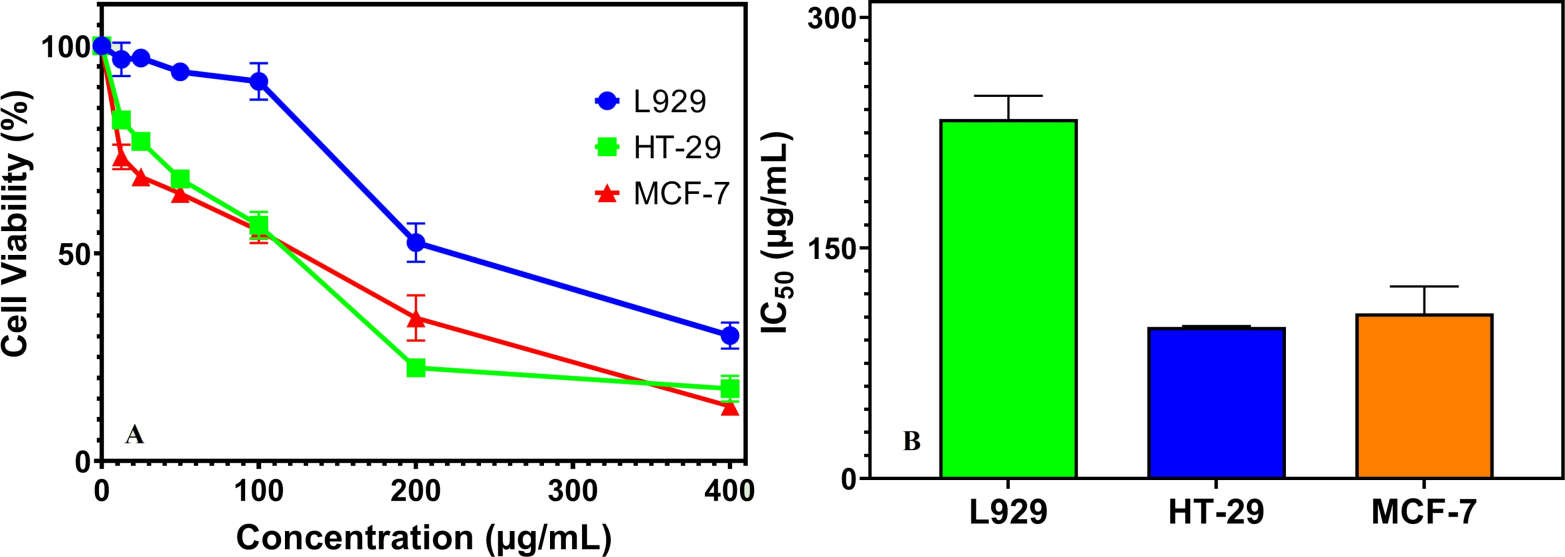
Cytotoxic effects of AgSbS_2_ NCs on different cell lines (a), IC_50_ values of AgSbS_2_ NCs on different cell lines (b).

Alamar Blue assay was used to evaluate the cell viability of the synthesized NCs (12.5–400 μg/mL) on HT-29 colon cancer, MCF-7 breast cancer, and L929 fibroblast cells through cellular activity. The cytotoxicity of the synthesized NCs on these cell lines after 24 h of treatment is revealed in Figure 4. The NCs induced a 3–26% inhibition of cell growth even at the lowest applied concentration (12.5 µg/mL), and a proportional increase in the percentage of cell inhibition was observed with escalating concentrations. Hence, the findings reveal the dose-dependent interaction of the nanoparticles with tested cell lines. The synthesized NCs had a significant toxic impact on the HT-29 cell line, causing the most effective cell inhibition, destroying 18% of HT-29 cells at the lowest concentration (12.5 µg/mL) and approximately 83% (IC_50_ = 98.67 ± 0.52 μg/mL) at the highest concentration (400 μg/mL). In addition, AgSbS_2_ NCs showed a significant cytotoxicity against MCF-7 breast cancer cells, exhibiting a significant inhibitory effect with 87% cell inhibition (IC_50_ = 107.41 ± 17.53 μg/mL) at the highest concentration tested (Figure 4). The synthesized NCs caused lower cell inhibition on healthy cell lines (L929) (IC_50_ = 233.83 ± 15.07 μg/mL) compared to cancer cell lines, demonstrating 69% lethality at 400 μg/mL. Therefore, the synthesized NCs in this study exhibit an enhanced efficacy in inducing cytotoxicity in cancerous cells. Similar results were reported for other synthesized nanoparticles [44–45]. Cell inhibition in cell lines can be attributed to intracellular accumulation of nanoparticles leading to oxidative stress-induced apoptosis and necrosis [46]. The findings of this investigation demonstrate that NCs exhibit a substantially lower inhibitory effect on healthy cell lines compared to their pronounced impact on malignant cell lines, thereby establishing a differential selectivity profile. This differential efficacy suggests considerable potential for NCs to function as targeted therapeutic agents in cancer treatment, demonstrating promising selectivity towards cancerous cells. A comprehensive understanding of the fundamental mechanisms governing this selective activity against cancer cell lines necessitates further investigation. Elucidating the specific molecular interactions and signaling pathways between NCs and malignant cells may provide an alternative therapeutic avenue for the development of targeted cancer therapies by leveraging this inherent selectivity.

Although direct mechanistic assays such as ROS quantification or apoptosis marker analysis were not performed in this study, previous research on silver-based nanomaterials offers valuable insights into potential pathways behind the observed cytotoxicity. Several studies have reported that AgNPs cause intracellular accumulation, which triggers the overproduction of ROS. This oxidative stress can result in mitochondrial dysfunction, lipid peroxidation, DNA fragmentation, and the activation of apoptotic signaling pathways. For example, green-synthesized silver nanoparticles were shown to induce ROS production and caspase-3/9 activation, leading to apoptosis in MCF-7 breast cancer cells [47]. Similarly, ROS-mediated cytotoxicity and apoptosis have been demonstrated in HepG2 liver carcinoma cells treated with AgNPs, supporting oxidative stress as a key factor in nanoparticle-induced cell death [48]. Therefore, it is plausible that the cytotoxic effects of AgSbS_2_ NCs observed in this study are at least partially mediated through ROS-dependent apoptotic mechanisms. Future investigations focusing on direct ROS measurement and apoptosis marker assays (e.g., Annexin V/PI staining, caspase-3 activation) will be crucial for validating and further elucidating these mechanistic pathways. The surface charge and zeta potential of AgSbS_2_ NCs, which strongly influence their colloidal stability and cellular uptake, are expected to be within the range typically reported for Ag-based chalcogenides exhibiting stable dispersions.

#### Antioxidant activity of AgSbS_2_ NCs

**Figure 5:**
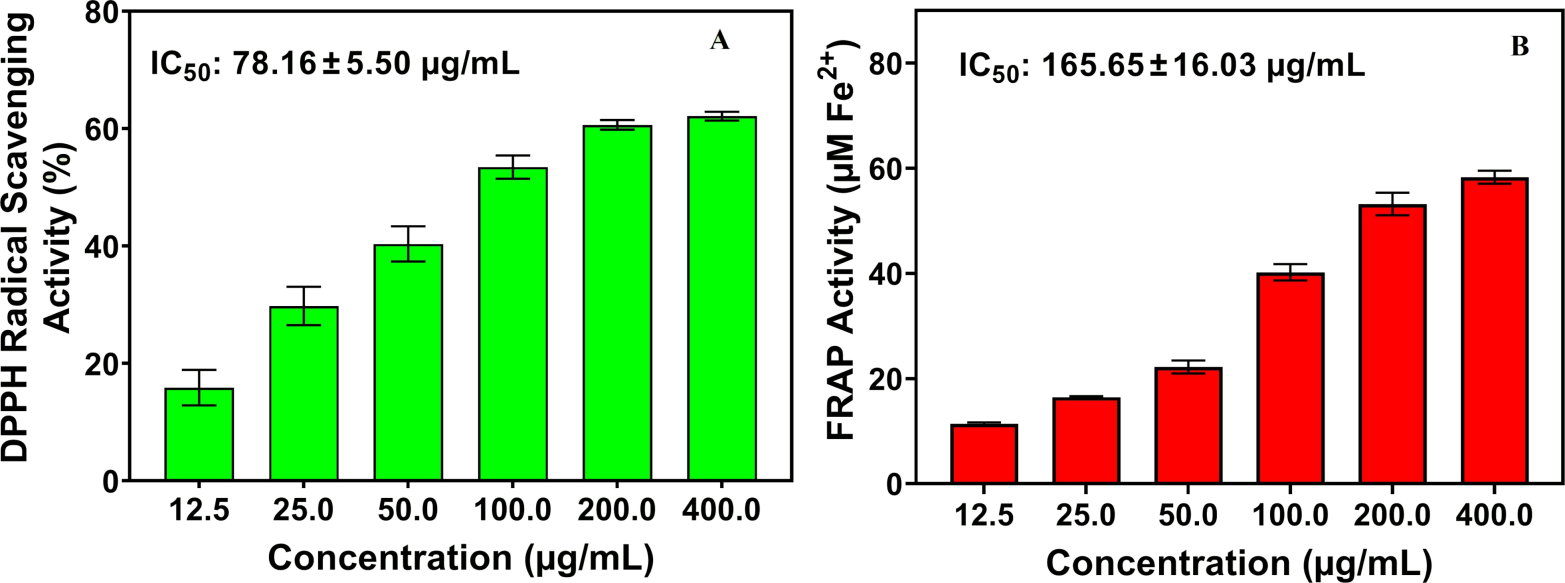
DPPH radical scavenging activity of AgSbS_2_ NCs (a), FRAP activity of AgSbS_2_ NCs (b).

The DPPH radical scavenging activity of AgSbS_2_ NCs in the assay using ascorbic acid as a positive control is revealed in Figure 5. The synthesized NCs demonstrated significant antioxidant activity, as assessed by the DPPH assay. The effectiveness of the nanoparticles increased with concentration. Notably, the AgSbS_2_ nanostructures exhibited the highest inhibition percentage (62.11%) at the highest tested concentration (400 μg/mL), with an IC_50_ value of 78.16 ± 5.50 µg/mL (*p* < 0.05) (Figure 5). These findings suggest that AgSbS_2_ NCs possess potent free radical scavenging abilities.

The antioxidant properties of nanoparticles are exceptional due to their unique size-dependent characteristics, particularly their large surface area-to-volume ratio and enhanced surface reactivity [30]. These features, combined with the tunable surface chemistry of nanoparticles, allow for efficient interactions with free radicals and facilitate electron transfer reactions, resulting in robust antioxidant activity [49]. This activity involves scavenging free radicals by donating electrons, thereby stabilizing these reactive species and mitigating potential cellular damage. The primary mechanism of action for the synthesized nanoparticles in this study is likely to involve electron donation to the DPPH radical [50].

Consistent with the DPPH results, the FRAP assay results (Figure 5) showed a concentration-dependent increase in ferric-reducing antioxidant power. At the lowest concentration (12.5 µg/mL), the activity was ≈8 µM Fe^2+^ equivalent, climbing steadily to ≈58 µM Fe^2+^ equivalent (IC_50_ = 165.65 ± 16.03 µg/mL) at 400 µg/mL. These results demonstrate that AgSbS_2_ NCs possess measurable electron-donating potential, with moderate reducing strength under the assay conditions.

In the realm of nanomaterials, enhanced reducing power is often attributed not only to intrinsic redox-active moieties but also to high surface area, quantum size effects, and effective exposure of surface facets to the reaction medium. Green-synthesized nanoparticles in various studies have consistently shown elevated FRAP responses compared to their bulk counterparts, due to improved electron transfer kinetics at the nano–bio interface [51–52]. When considered alongside DPPH scavenging data, the FRAP outcome supports a dual-mechanism antioxidant model (both radical quenching and electron transfer). This suggests that the surface chemistry of AgSbS_2_ NCs effectively mediates both redox and radical-based pathways, making them promising candidates for applications requiring moderate reducing capacity.

## Conclusion

Ag-based materials, which are well known for their antibacterial properties, have been the subject of many studies. Therefore, in this study, AgSbS_2_ NCs belonging to the Ag-based chalcogenide material family were produced using the hot-injection method to examine their biological properties. As a result of the performed characterizations, it was revealed that monodisperse AgSbS_2_ NCs were obtained in high purity and crystallinity. A remarkable antibacterial activity of the synthesized AgSbS_2_ NCs was identified by exhibiting an ability to suppress microbial growth against pathogenic microorganisms. Moreover, the strong anticancer effects of these NCs against HT-29 and MCF-7 cancer cell lines suggest promising alternative strategies for the therapeutic treatment of colon and breast cancers, while their remarkable antioxidant properties indicate that they may exhibit versatile biofunctional characteristics relevant to biomedical applications. Despite these promising findings, the results are limited to in vitro assays. Future work should therefore prioritize in vivo validation, mechanistic studies, and surface engineering strategies to enhance biocompatibility and therapeutic specificity. Integration with emerging nanomedicine approaches such as theranostics, controlled drug delivery, and precision oncology could further advance the translational potential of AgSbS_2_ NCs.

## Data Availability

All data that supports the findings of this study is available in the published article and/or the supporting information of this article.
